# Impaired Exercise Performance and Skeletal Muscle Mitochondrial Function in Rats with Secondary Carnitine Deficiency

**DOI:** 10.3389/fphys.2016.00345

**Published:** 2016-08-10

**Authors:** Jamal Bouitbir, Patrizia Haegler, François Singh, Lorenz Joerin, Andrea Felser, Urs Duthaler, Stephan Krähenbühl

**Affiliations:** ^1^Department of Clinical Pharmacology and Toxicology, University Hospital BaselBasel, Switzerland; ^2^Department of Biomedicine, University of BaselBasel, Switzerland; ^3^Swiss Centre of Applied Human ToxicologyBasel, Switzerland; ^4^Fédération de Médecine Translationelle, Faculté de Médecine, Institut de Physiologie, Université de StrasbourgStrasbourg, France

**Keywords:** N-trimethyl-hydrazine-3-propionate, carnitine deficiency, exhaustive exercise, skeletal muscle, mitochondria, reactive oxygen species

## Abstract

**Purpose:** The effects of carnitine depletion upon exercise performance and skeletal muscle mitochondrial function remain largely unexplored. We therefore investigated the effect of N-trimethyl-hydrazine-3-propionate (THP), a carnitine analog inhibiting carnitine biosynthesis and renal carnitine reabsorption, on physical performance and skeletal muscle mitochondrial function in rats.

**Methods:** Male Sprague Dawley rats were treated daily with water (control rats; *n* = 12) or with 20 mg/100 g body weight THP (*n* = 12) *via* oral gavage for 3 weeks. Following treatment, half of the animals of each group performed an exercise test until exhaustion.

**Results:** Distance covered and exercise performance were lower in THP-treated compared to control rats. In the oxidative soleus muscle, carnitine depletion caused atrophy (–24%) and impaired function of complex II and IV of the mitochondrial electron transport chain. The free radical leak (ROS production relative to oxygen consumption) was increased and the cellular glutathione pool decreased. Moreover, mRNA expression of markers of mitochondrial biogenesis and mitochondrial DNA were decreased in THP-treated compared to control rats. In comparison, in the glycolytic gastrocnemius muscle, carnitine depletion was associated with impaired function of complex IV and increased free radical leak, whilst muscle weight and cellular glutathione pool were maintained. Markers of mitochondrial proliferation and mitochondrial DNA were unaffected.

**Conclusions:** Carnitine deficiency is associated with impaired exercise capacity in rats treated with THP. THP-induced carnitine deficiency is associated with impaired function of the electron transport chain in oxidative and glycolytic muscle as well as with atrophy and decreased mitochondrial DNA in oxidative muscle.

## Introduction

Carnitine, 4-trimethylamino-3-hydroxy butyric acid, is present in all mammalian tissues and is ingested by the diet or produced by endogenous biosynthesis from trimethyllysine (Bremer, 1983; Hoppel and Davis, 1986; Vaz and Wanders, 2002). The major carnitine body pool, accounting for >95% of the total body stores, is located in skeletal muscle (Hoppel and Davis, 1986; Krähenbühl et al., 2000; Spaniol et al., 2001). Carnitine is an obligatory intermediate for the transport of long chain fatty acids across this inner mitochondrial membrane and plays an essential role in cellular energy metabolism due to acylation of its β-hydroxyl group (Fritz and Mc, 1959; Bremer, 1983). Furthermore, carnitine is important for buffering the free pool of coenzyme A (CoASH). For instance, carnitine affects carbohydrate metabolism by stimulating glucose oxidation *via* reversing the inhibition of pyruvate dehydrogenase by accumulating acetyl-CoA (Roberts et al., 2002; Wall et al., 2011). Moreover, carnitine shifts the acyl-CoA to CoA-SH ratio in the direction of CoASH and is thereby involved in trapping acyl residues from peroxisomes and mitochondria (Brass and Hoppel, 1980; Friolet et al., 1994).

Low carnitine body stores can be primary as an inherited defect of the main carnitine transporter OCTN2 (Organic Cation/Carnitine Transporter 2) or secondary as a result of an excessive renal loss or diminished supply of carnitine and carnitine precursors. Primary carnitine deficiency is a genetic disorder of the cellular carnitine-transporter system associated with mutations in OCTN2 (Treem et al., 1988), a sodium-dependent, high affinity carnitine carrier with a high expression and activity in the renal proximal tubule (Nezu et al., 1999). Secondary carnitine deficiency is associated with reduced consumption of carnitine and carnitine precursors, e.g., in vegetarians (Stephens et al., 2011; Novakova et al., 2015), increased renal excretion of carnitine and acylcarnitines in patients with organic acidurias (Roe et al., 1984) or patients treated with drugs producing acyl-groups (Brass et al., 2003; Morand et al., 2012), or in patients on hemodialysis which removes carnitine from the circulation (Hiatt et al., 1992; Evans et al., 2000). Muscle carnitine deficiency, which can be a consequence of primary and secondary forms of carnitine deficiency, is associated with muscle weakness and fatigue (Treem et al., 1988; Hiatt et al., 1992), and possibly cardiomyopathy (Zaugg et al., 2003). A low muscle carnitine content is associated with impaired transport of long-chain fatty acids into the mitochondrial matrix and therefore reduced β-oxidation and accumulation of potentially toxic free fatty acids and acyl-CoAs (Bremer, 1983).

N-trimethyl-hydrazine-3-propionate (THP or mildronate) is a carnitine analog that has been used in certain countries as a cardioprotective agent. This compound was used to generate and characterize a rat model with secondary carnitine deficiency due to inhibition of OCTN2 and γ-butyrobetaine hydroxylase, the last enzyme of the carnitine biosynthesis pathway (Vaz and Wanders, 2002). THP treatment of rats for 2–3 weeks has been shown to reduce the carnitine content of liver, heart, plasma, and skeletal muscle by 70–80% (Spaniol et al., 2001). This decrease in the carnitine content was associated with liver steatosis (Spaniol et al., 2003), impaired myocardial function (Zaugg et al., 2003), and impaired contractile force of the extensor digitorum longus and atrophy of the soleus muscle in rats (Roberts et al., 2015).

While it is clear that THP-treated animals with secondary carnitine deficiency have an impaired contractile function of specific muscles (Roberts et al., 2015), it is currently unclear, whether this impairs the exercise capacity of these rats. Furthermore, it is currently also not known whether carnitine deficiency affects mitochondrial function and/or mitochondrial biogenesis. In athletes, long-term treatment with high oral doses of carnitine was associated with improved exercise capacity and mitochondrial function (Huertas et al., 1992).

Regarding the established effects of carnitine on skeletal muscle energy metabolism (Stephens et al., 2007), and the currently available data in THP rats (Roberts et al., 2015), we hypothesized that rats with THP-induced secondary carnitine deficiency may have an impaired exercise capacity and impaired skeletal muscle mitochondrial function. In the present study, we therefore performed an exhaustive exercise test (in order to test mainly the buffer function of carnitine) in rats treated with THP and examined mitochondrial function in two functionally different muscle groups, the oxidative soleus and the glycolytic gastrocnemius muscles. The main aim of the study was to improve our knowledge about the consequences of secondary carnitine deficiency on skeletal muscle function and physical performance.

## Materials and methods

### Ethical approval

*In vivo* experiments were performed in accordance with the National Institute of Health guide for the care and use of Laboratory animals (NIH Publication No. 8023, revised 1978), and were approved by the Cantonal Veterinary Department of Basel, Switzerland (License 1745). 24 male Sprague Dawley rats, weighing ~250 g (6 weeks), were obtained from Janvier Labs (Saint-Berthevin, France), and were acclimatized to the laboratory 1 week prior to the start of the study. The animals were housed in a temperature (20–22°C) and light (12 h light − 12 h dark cycle) controlled animal facility.

### Compound and treatment

**Figure 1:**
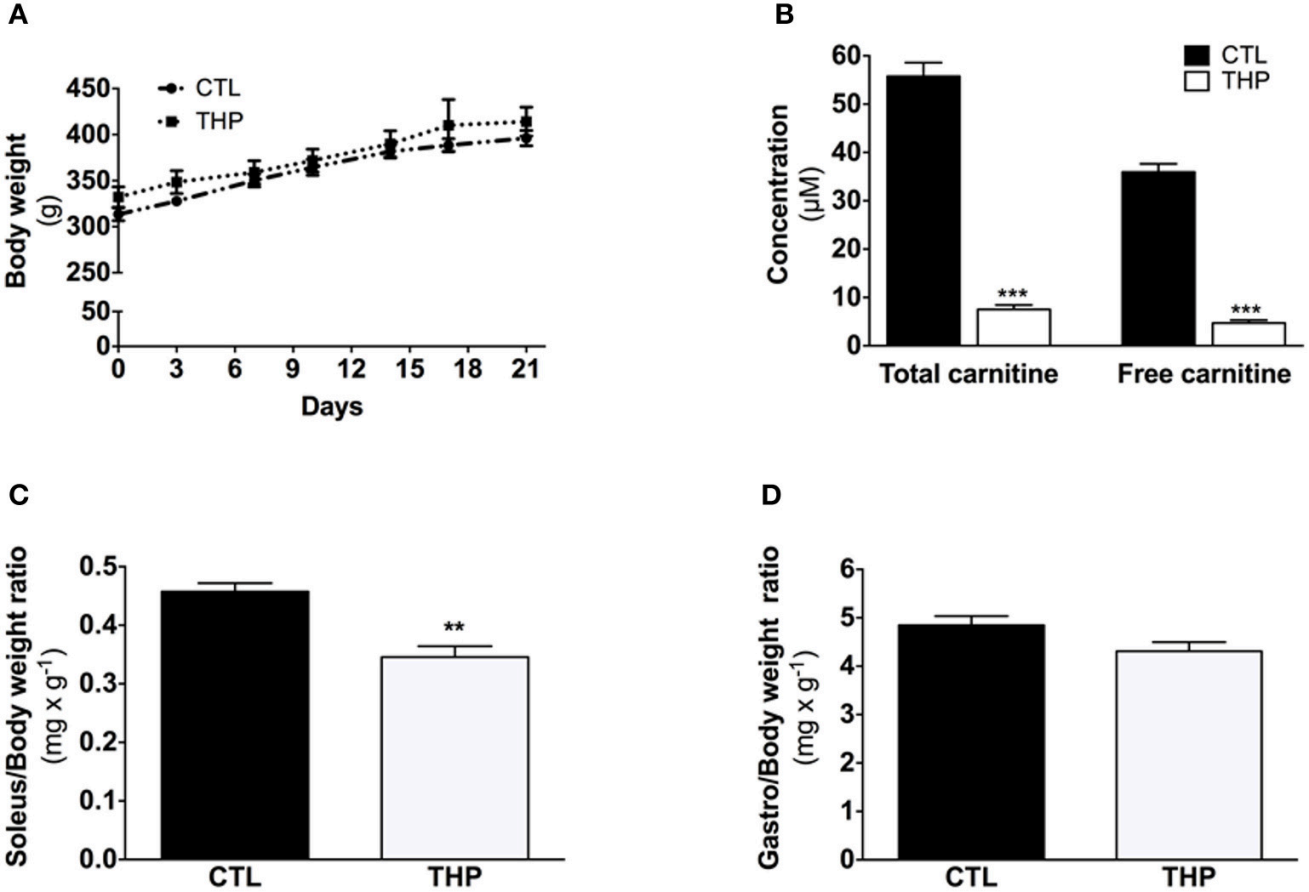
**Characterization of the animals**. **(A)** Evolution of body weight during the treatment. **(B)** Plasma total and free carnitine in rats treated or not with THP. **(C)** Soleus and **(D)** gastrocnemius weight relative to the body weight. Data are represented as mean ± SEM of 6 animals per group. ^**^p < 0.01 and ^***^p < 0.01 compared to CTL group.

THP (N-trimethyl-hydrazine-3-propionate) was prepared by ReseaChem GmbH (Burgdorf, Switzerland) as described previously (Spaniol et al., 2001). After 1 week of acclimatization, 24 rats were randomly divided into two groups as follows: (1) animals treated with water (CTL, *n* = 12); (2) animals treated with THP (20 mg × 100 g^−1^ body weight × day^−1^ for 21 days) (THP; *n* = 12). THP was dissolved in water and the animals were treated for 3 weeks by oral gavage. Half of the animals of each group performed an exhaustive exercise before sacrifice. Water was provided *ad libitum* throughout the study. Food consumption and changes in body weight were examined every 3 days (Figures 1A,B). Based upon the findings of our previous study, the rats received a standard chow (Kliba Futter 3433, Basel, Switzerland) *ad libitum* (carnitine content 16.9 nmol × g^−1^), as the effect of carnitine-deficient food upon carnitine homeostasis is minor in rats treated with THP (Spaniol et al., 2001).

### Treadmill exercise

After 3 weeks of treatment, the rats were submitted to an exhaustive exercise on a treadmill with previous acclimatization to the apparatus (Treadmill Control, Panlab®, Barcelona, Spain). Two days before the sacrifice, rats were accustomed to the treadmill in a daily session of 5-min training at a speed of 40 cm × s^−1^ with a slope of +5°. On the day of sacrifice, the starting speed was 40 cm × s^−1^ with a slope of +5°; after 40 min, the speed was increased by 5 cm × s^−1^ every 2 min until exhaustion (Bouitbir et al., 2011). The rear of the treadmill was equipped with low-voltage, electric stimulating grids, to encourage the rats to run. The grid delivered 0.4 mA at a frequency of 2 Hz, which caused an uncomfortable shock but without injuring the animal. Exhaustion was defined as immobility for more than 5 s on the end lane grid despite electric stimulation (Bouitbir et al., 2011). Blood lactate concentrations were determined in a sample collected from the tip of the tail before, immediately after and 15 min after exercise using a lactate pro-LT device (Lactate Pro LT-1710, ARKRAY®).

Vertical mechanical work and power of the exercise session were calculated according to Isner-Horobeti et al. (2014):

(1) $$\begin{array}{rcl} \text{Vertical mechanical work }\left(\text{J}\right) & = & \text{height}\times \text{m}\times \text{g} \end{array}$$

(2) $$\begin{array}{rcl} \text{Vertical mechanical power }\left(\text{W}\right) & = & \text{vertical mechanical work}/\text{t} \end{array}$$

Where: J = Joule; W = Watt; t = running time in s; height in meter calculated with the Pythagorean theorem; m = animal body weight in kg, and g = gravitational constant in m × s^−2^.

### Biological sample collection

After exhaustion, the rats were anesthetized with an intraperitoneal application of ketamine (160 mg × kg^−1^) and xylazine (20 mg × kg^−1^). The soleus (oxidative muscle composed predominantly with type I fibers), and the superficial part of the gastrocnemius (glycolytic muscle composed predominantly with type II fibers) were excised and conserved in ice-cold BIOPS buffer (10 mM Ca-EGTA buffer, 0.1 μM free calcium, 5.77 mM ATP, 6.56 mM MgCl_2_, 20 mM taurine, 15 mM phosphocreatine, 0.5 mM dithiothreitol, and 50 mM K-MES, pH 7.1) until analysis. A part of the muscle samples was frozen in liquid nitrogen immediately after excision for further analysis.

Blood was obtained by intracardiac puncture or tail incision and collected into heparin-coated tubes. Plasma was separated by centrifugation at 3000 g for 15 min. Plasma and muscle samples were kept at −80°C until analysis.

### Plasma parameters

Creatine kinase activity in plasma was determined before and after exhaustive exercise with a kit according to the supplier's instructions (BioAssay Systems, Hayward, CA, USA). Free and total carnitine concentrations were determined as described previously (Morand et al., 2013).

### High-resolution measurement of mitochondrial respiration

Mitochondrial respiration measured *in situ* allows the characterization of functional mitochondria in their normal intracellular position and assembly, preserving essential interactions with other organelles. Mitochondrial oxygen consumption was studied in saponin-skinned fibers (Kuznetsov et al., 2008). Fibers were separated under a binocular microscope in BIOPS buffer at 4°C. After dissection, fibers were transferred into relaxing BIOPS buffer containing 50 μg × mL^−1^ saponin and incubated at 4°C for 30 min while shaking to complete permeabilization of the sarcolemma. Permeabilized fibers were then washed in BIOPS buffer for 10 min under intense shaking to completely remove saponin. Before oxygraphic measurements, the fibers were washed twice for 5 min in MiR05 buffer (EGTA 0.5 mM, MgCl_2_ 3 mM, taurine 20 mM, KH_2_PO_4_ 10 mM, HEPES 20 mM, D-sucrose 110 mM, BSA essentially fatty acid free 1 g/L, and lactobionic acid 60 mM) to remove any traces of high-energy phosphates. All oxygen measurements were performed at 37°C with an Oxygraph 2k apparatus equipped with the Datlab software (OROBOROS, Innsbruck, Austria).

Wet weight of the fibers (2–3 mg) was determined after permeabilization, which reduces osmotic variations of the water content as described (Pesta and Gnaiger, 2012).

Loosely connected fiber bundles were placed for 5 s onto dry filter paper. During this time period, we wiped off any liquid from the tip of the forceps with another filter paper. Then, we took the sample from the filter paper by a forceps and removed any trace of water on the surface on a dry area of the filter paper before weighing the sample. Immediately weight recording, the sample was transferred into 2.0 mL of MiR05 buffer and analyzed by high-resolution oximetry.

The rate of basal respiration was measured with the complex I substrates glutamate (10 mM) and malate (2 mM), before state 3 respiration was induced with ADP (2 mM). Since oxygen is the electron acceptor, enzyme complexes I, III and IV are used under these conditions. After inhibition of complex I with rotenone (0.5 μM), respiration was restored with addition of the complex II substrate succinate (10 mM). Under these conditions, enzyme complexes II, III, and IV were assessed. To verify the integrity of the outer mitochondrial membrane, cytochrome c (10 μM) was added. Inhibition of complex III with antimycin (2.5 μM) was followed by the addition of artificial substrates for complex IV, TMPD (0.5 mM) and ascorbate (2 mM). We performed the experiments in duplicate. Respiration rates are expressed in picomoles O_2_ × s^−1^ × mg^−1^ wet weight.

### Activity of individual enzyme complexes of the electron transport chain

Spectrophotometric analysis of the activity of individual enzyme complexes was performed as described by Spinazzi et al. (2012). In brief, frozen muscle samples were thawed and homogenized in water.

Complex I + III assay was performed in an assay mixture composed of (final concentrations) 50 mM potassium phosphate, 1 g/L BSA, 0.3 mM KCN, 50 μM ferricytochrome c, and 0.5 mM reduced nicotinamide adenine dinucleotide (NADH), pH 7.4. The increase in absorbance was followed at 550 nm. The rotenone-sensitive complex I + III activity was determined by subtracting the activity of identical wells containing in addition 1 μM rotenone.

Complex II activity was measured in an assay mixture containing (final concentrations) 50 mM potassium phosphate, 1 g/L BSA, 0.1 mM Na_2_EDTA, 3.75 mM sodium azide, 0.3 mM KCN, 20 mM succinate, 100 μM dichlorophenolindophenol (DCIP), pH 7.2. Changes in absorbance were followed at 600 nm. Malonate sensitive activity was calculated by subtracting the activity of identical wells containing 10 mM malonate.

Complex IV activity was measured in an assay buffer containing (final concentrations) 50 mM potassium phosphate and 17 μM of freshly prepared ferrocytochrome c, pH 7.4. Changes in absorbance were followed at 550 nm. Specific complex IV was calculated by subtracting the activity of identical wells containing 0.3 mM KCN.

Complex activities were determined on a Tecan M200 Pro Infinity plate reader (Männedorf, Switzerland) using a 96-well plate. Values were normalized to citrate synthase activity.

### Citrate synthase (CS) activity

Pieces of frozen muscle (5–10 mg wet weight) were homogenized with a vibrating microbead homogenizer (Mikro-Dismembrator, Sartorius®, Palaiseau, France) in a ratio (W/V) of 1/20 with a buffer containing 5 mM HEPES, 1 mM EGTA and 1 mM DTT, pH 8.7. The homogenate was then supplemented with 0.1% Triton X-100 and incubated on ice for 1 h. After centrifugation for 5 min at 3000 rpm, CS activity was determined in the supernatant by spectrophotometry (Tecan M200 Pro Infinity plate reader, Männedorf, Switzerland) using a 96-well plate as described by (Srere, 1969). Values were reported in μmol × min^−1^ × g^−1^ of wet weight.

### H_2_O_2_ production rates in permeabilized muscle fibers

Mitochondrial H_2_O_2_ production rates by permeabilized muscle fibers were determined as described previously using Amplex Red reagent (Invitrogen, Switzerland; Anderson and Neufer, 2006). Change in fluorescence per second (ΔF) was measured continuously with a spectrofluorometer (Fluoromax 4 Jobin Yvon®, Edison, New Jersey, USA). First, background ΔF (reactants only) was established before the reaction was initiated by addition of 2–3 mg permeabilized muscle fibers (from soleus or gastrocnemius to 600 μl of buffer Z containing K-methane sulfonate 110 mM, KCl 35 mM, EGTA 1 mM, K_2_HPO_4_ 10 mM, and MgCl_2_ 3 mM with Amplex Red 5 μM and horseradish peroxidase 0.3 U/ml (Invitrogen, Switzerland). The following additions were then made sequentially: the complex I substrate glutamate-malate (5 and 2.5 mM, respectively), the complex II substrate succinate (5 mM), the complex I blocker rotenone (0.5 μM) and the complex III blocker antimycin A (2.5 μM).

The rates of H_2_O_2_ production were calculated using a standard curve established with the same experimental conditions, except that fibers were absent. The background activity was subtracted from the activity obtained in the presence muscle fibers. H_2_O_2_ measurements were expressed in pmoles × min^−1^× mg^−1^ wet weight.

### Mitochondrial free radical leak (FRL)

H_2_O_2_ production and O_2_ consumption were measured simultaneously in the same sample in the presence of glutamate + malate, rotenone, succinate, and ADP allowing to quantify the parameter under respiration supported by the complex II donor succinate under state 3 conditions. This allowed the calculation of the fraction of electrons out of sequence, which reduce O_2_ to ROS in the respiratory chain (the percentage of free radical leak) instead of reaching cytochrome oxidase to reduce O_2_ to water. Because two electrons are needed to reduce one mole of O_2_ to H_2_O_2_, whereas four electrons are transferred in the reduction of one mole of O_2_ to water, the percent of FRL was calculated according to Anderson and Neufer (2006) as the rate of H_2_O_2_ production divided by twice the rate of O_2_ consumption, and then the result was multiplied by a factor of 100.

### Tissue GSH content

The GSH (reduced glutathione) content was determined using the luminescent GSH-Glo Glutathione assay (Promega, Wallisellen, Switzerland) according to the manufacture's manual. In brief, muscle tissue (10 mg) was homogenized in 1 mL PBS with 2 mM EDTA with a Mikro-Dismembrator for 1 min at 3000 rpm (Sartorius Stedim Biotech, Göttingen, Germany). After centrifugation, 50 μL supernatant was added to 50 μl GSH-Glo Reagent in a 96-well plate and incubated for 30 min. Afterwards, 100 μl Luciferin Detection Reagent was added to each well and the luminescence was measured after 15 min using a Tecan M200 Pro Infinity plate reader (Männedorf, Switzerland). The GSH standard curve was performed and the results were expressed in μmol × g^−1^ of wet weight

### Muscle parameters

Muscle tissue (50 mg) was homogenized with a Mikro-Dismembrator for 1 min at 2000 rpm (Sartorius Stedim Biotech, Göttingen, Germany). Muscle glycogen content was analyzed in alkaline (NaOH 0.1 mM) muscle extracts according to Harris et al. (1974). The lactate content was analyzed according to Olsen (1971).

### Quantitative real-time polymerase chain reaction (q-RT-PCR)

Total RNA was extracted from muscle using a commercially available TRIzol Reagent kit (Invitrogen, Basel, Switzerland). Briefly, 50 mg of muscle were removed from the freezer and immediately immersed in 1 ml of TRIzol reagent. The aqueous and organic phases were separated using 200 μl of chloroform. Total RNA was precipitated using 600 μl of isopropyl alcohol, washed twice with ethanol, redissolved in RNAse free H_2_O and stored at −80°C. The concentration and purity of the RNA were determined using a Nanodrop (Thermo Scientific, Wohlen, Switzerland) by measuring the absorbance at 260 and 280 nm. The reverse transcription (RT) was performed on 1 μg of total RNA using a commercially available kit (Invitrogen, Life Technologies, Switzerland). RT was performed in a thermal cycler with a profile of 65°C for 5 min, 42°C for 50 min, and 72°C for 15 min. All samples were run together. Following RT, samples were stored at −20°C until analysis. Real—time PCR measurement of individual cDNAs was performed in triplicate using SYBR green dye (Roche Diagnostics, Rotkreuz, Basel) containing 10 μM of each primer (sense and antisense). Primer sequences were designed using information contained in the public database in the GeneBank of the National Center for Biotechnology Information. The sequences of primer sets used are listed in Table 1. Amplification efficiency of each sample was calculated as previously described (Ramakers et al., 2003), and relative mRNA expression levels were calculated using the ΔΔCT method with beta actin gene as internal control.

**Table 1:** **Primer sequences used for quantitative Real-Time PCR amplification**.

**Target gene**	**Forward primer 5′ → 3′**	**Accession number**
	**Reverse primer 5′ → 3′**	
PGC-1α	GCTGGACACTGGACTTCCTC	NC 005100
	GAGGACTCCAGCCACAAAGA	
PGC-1β	CGACTTTGCAGAGATGTCCA	NC 005112
	CCTGAAGAGTTCCTCCACCA	
TFAm	GAAAGCACAAATCAAGAGGAG	NM 031326
	CTGCTTTTCATCATGAGACAG	
SOD1	AGATGACTTGGGCAAAGGTG	NM 017051
	CAATCCCAATCACACCACAA	
SOD2	CTGGACAAACCTGAGCCCTA	NM 017051
	GAACCTTGGACTCCCACAGA	
Beta actin	TTGCTGACAGGATGCAGAAG	BC 166732
	CAGTGAGGCCAGGATAGAGC	
Cyt b	GCAGCTTAACATTCCGCCCAATCA	J01436
	TACTGGTTGGCCTCCGATTCATGT	
Pyruvate kinase	TGTGGGTGATCTGGTGATTGTGGT	NM 012624
	AGGCATTTCAGGATACGCTCAGCA	

### Mitochondrial DNA content

To measure the mitochondrial DNA content, we determined the ratio of one mitochondrial gene to a nuclear gene using quantitative real-time RT-PCR as described previously with some modifications (Pieters et al., 2013). Total DNA was extracted using the DNeasy Blood and Tissue Kit (Qiagen) following the manufacturer's instructions (Quick-Start Protocol). The concentration of the extracted DNA was measured spectrophotometrically at 260 nm with the NanoDrop 2000 (Thermo Scientific, Wohlen, Switzerland). Afterwards, DNA was diluted in RNase free water to a final concentration of 10 ng × μL^−1^. The analysis of the mitochondrial cytochrome b and the nuclear pyruvate kinase genes purchased from Microsynth (Balgach, Switzerland) was performed using SYBR Green real-time PCR (Roche Diagnostics, Rotkreuz, Switzerland) on an ABI PRISM 7700 sequence detector (PE Biosystems, Rotkreuz, Switzerland). Relative amounts of nuclear and mitochondrial DNA were determined by comparison of the amplified and quantified DNA from pyruvate kinase and cytochrome b (Primer sequences in Table 1). The amount of mitochondrial DNA was also normalized to citrate synthase activity.

### Statistical analysis

Data is expressed as mean ± SEM. Comparisons of two groups were performed using the unpaired two-tailed Student's *t*-test. Multiple groups were compared using one-way ANOVA followed by a Tukey post-test. The software used was Prism version 6 (Graph Pad Software, San Diego, CA). Statistical significance was set at ^*^p < 0.05.

## Results

### Animal data

As shown in Figure 1A, the body mass was similar between THP-treated and CTL rats during the 21 days of chow feeding. Each group linearly gained ~80 g body weight during the course of the study. Daily food consumption was also not different between the CTL and THP-treated rats (data not shown). The total carnitine concentration was 7.5 ± 0.9 and 55.7 ± 2.8 μmol × L^−1^ and the plasma free carnitine concentration 4.7 ± 0.6 and 35.9 ± 1.6 μmol × L^−1^ in THP-treated and control rats, respectively (Figure 1B).

### Skeletal muscle weights

The absolute muscle weight of tendon-to-tendon excised oxidative soleus muscle was reduced by 24% in the THP-treated when compared to CTL rats (143 ± 6 vs. 187 ± 7 mg wet muscle; *p* < 0.01; data not shown). This decrease in muscle weight was maintained when the soleus weight was expressed relative to the body weight (Figure 1C). In contrast, no significant reduction in absolute weight (1780 ± 60 vs. 1970 ± 60 mg wet muscle in THP-treated and CTL rats, respectively; data not shown) or muscle weight relative to body weight (Figure 1D) was observed for the mainly glycolytic gastrocnemius muscle of THP-treated vs. CTL rats.

### Exhaustive exercise

**Figure 2:**
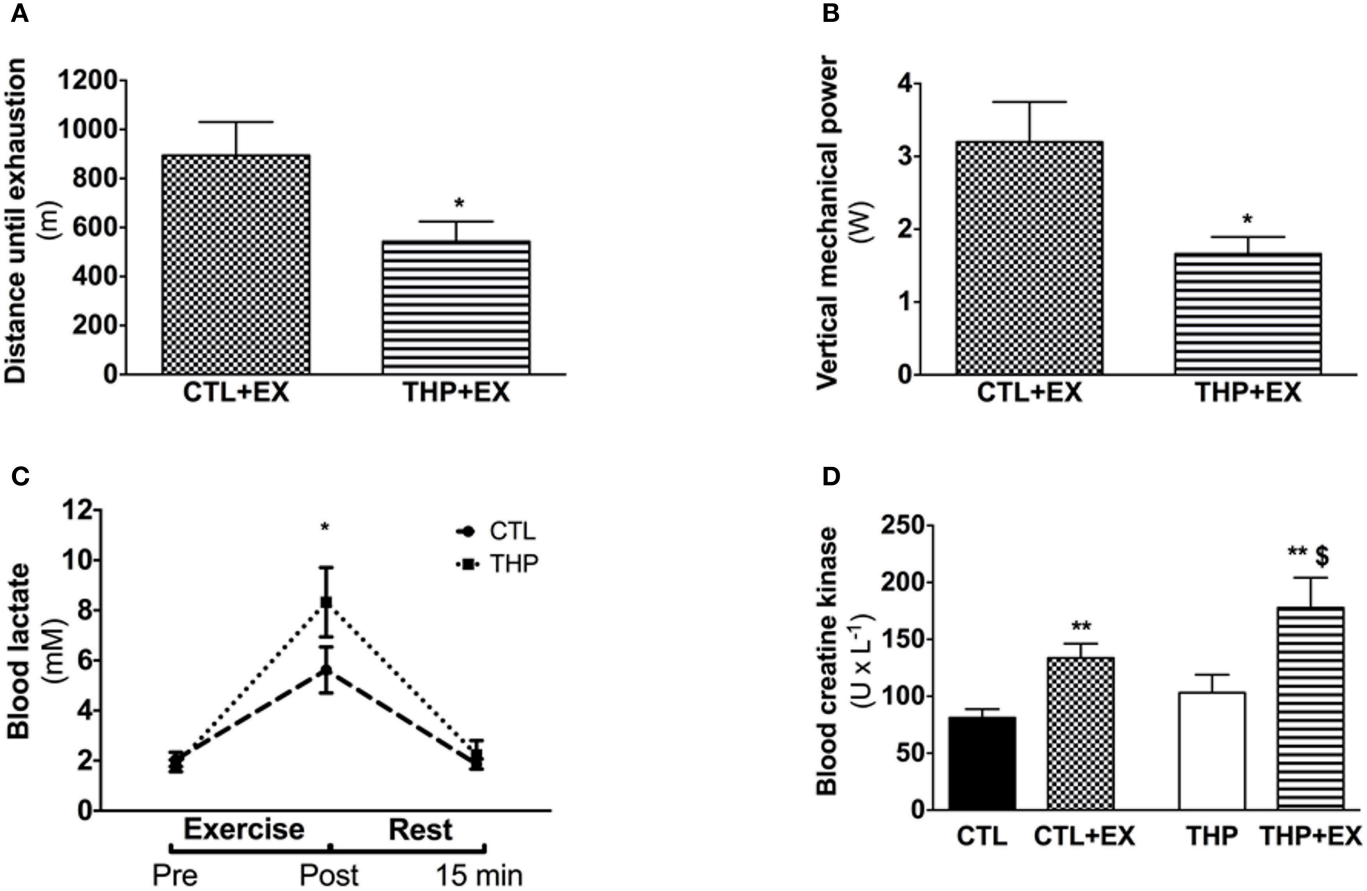
**Endurance test and plasma analysis**. **(A)** Endurance, evaluated by the average distance run until exhaustion on a treadmill performed in rats treated or not with THP and **(B)** vertical mechanical power calculated according the equation described in the methods. **(C)** Blood lactate concentration before, after and further 15 min rest following the exercise **(D)** blood creatine kinase measured in rats without exercise and after acute exercise in rats treated or not with THP. Data are represented as mean ± SEM of 6 animals per group. ^*^p < 0.05 and ^**^p < 0.01 compared to CTL group; ^$^*p* < 0.05 compared to CTL + EX group.

We performed an exhaustive treadmill exercise in a subgroup of THP-treated and CTL rats. As shown in Figure 2A, the distance that was covered by animals treated with THP was lower compared to the untreated animals (−39%; *p* < 0.05; Figure 2A). Since the animals should have covered 960 m during the first 40 min (speed of the treadmill 40 cm × s^−1^), the data show that the majority of the animals of both groups were exhausted already before 40 min (before increasing the speed of the treadmill). When we calculated the vertical mechanical power of the animals, we observed a significant decrease in the THP group compared to the CTL group (−42%; *p* < 0.05; Figure 2B).

### Energy metabolism parameters in plasma and skeletal muscle at rest and after exhaustive exercise

As shown in Figure 2C, the plasma lactate concentrations increased significantly with exercise in both treatment groups, indicating that the rats exercised above the lactate threshold. The plasma lactate concentration immediately after exercise was significantly higher in the THP compared to the control group (*p* < 0.05). After 15 min of recovery, the plasma lactate concentration had almost reached pre-exercise values in both treatment groups without a significant difference between the groups. As expected, the plasma creatine kinase activity increased significantly in both THP-treated and CTL rats with exercise (Figure 2D). After exercise, the plasma creatine kinase activity was significantly higher in THP-treated as compared to CTL rats.

### Energy parameters in skeletal muscles

**Figure 3:**
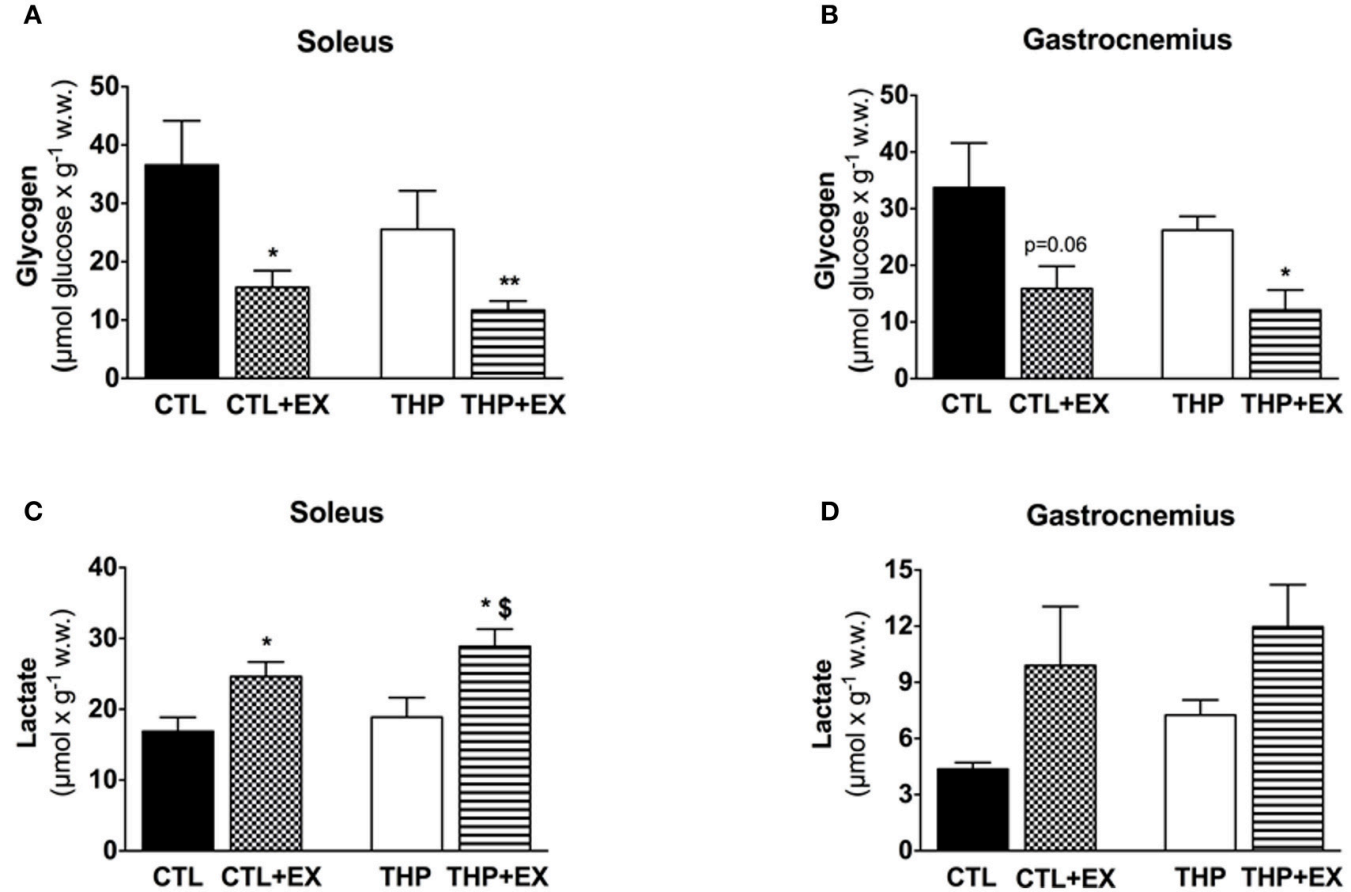
**Skeletal muscle metabolites at rest and after exercise**. Glycogen content measured as described in the methods in **(A)** soleus and **(B)** gastrocnemius muscles from groups. Lactate content measured as described in the methods in **(C)** soleus and **(D)** gastrocnemius muscles. Data are represented as mean ± SEM of 6 animals per group. ^*^p < 0.05 and ^**^p < 0.01 compared to CTL group; ^$^*p* < 0.05 compared to CTL + EX group.

Before exercise, the glycogen content was numerically lower in soleus and gastrocnemius muscle of THP-treated compared to CTL rats, but without reaching statistical significance (Figures 3A,B). Exhaustive exercise was associated with a significant decrease in the glycogen content in soleus and gastrocnemius muscle of both THP-treated and CTL rats, but there was no significant difference between the two groups. The lactate content in soleus significantly increased in THP-treated and CTL rats with exercise, reaching significantly higher values for THP-treated compared to CTL rats (Figure 3C). In the gastrocnemius, the lactate content increased in both THP-treated and CTL rats with exercise but without a significant difference between the two groups (Figure 3D).

### High-resolution measurement of mitochondrial respiration

**Figure 4:**
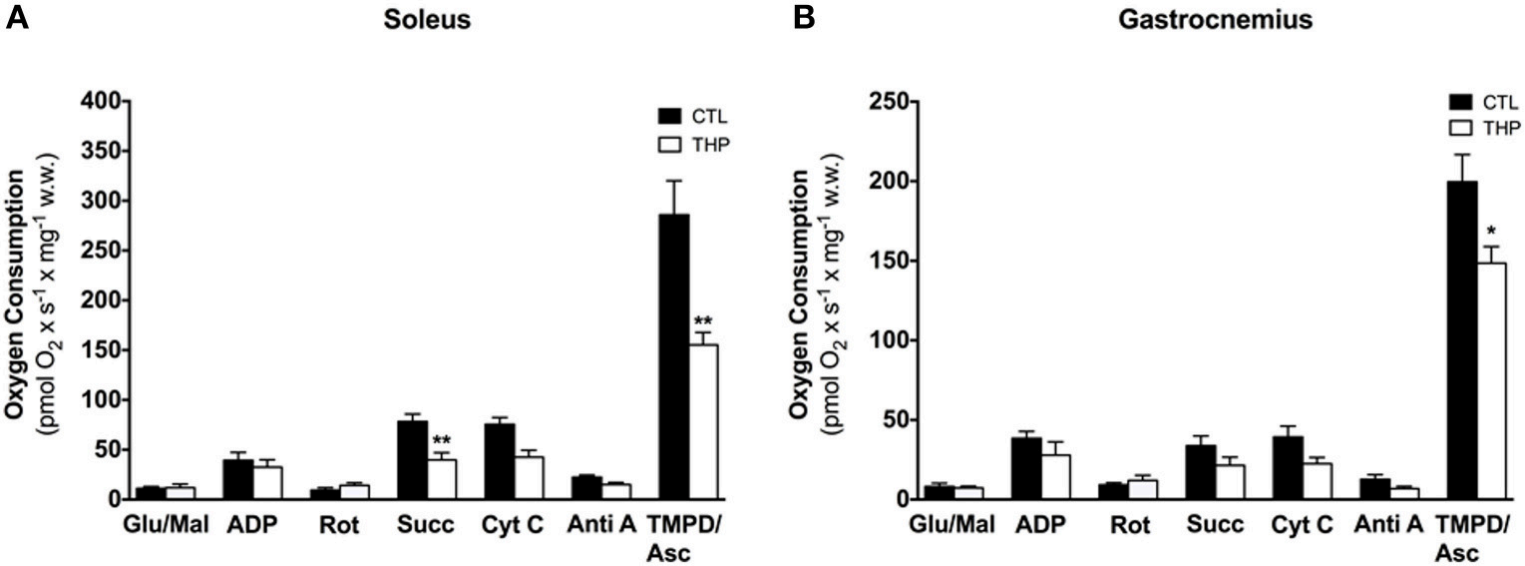
**Respiratory capacity in permeabilized muscle fiber mitochondria**. Oxygen consumption was measured in saponin-permeabilized muscle fibers with glutamate and malate as substrates in **(A)** soleus and **(B)** gastrocnemius. Data are represented as mean ± SEM of 5–6 animals per group. ^*^p < 0.05 and ^**^p < 0.01 compared to CTL group.

In order to find out possible reasons for the reduced physical performance of THP-treated rats, we determined the mitochondrial respiration of the electron transport chain in mitochondria of soleus and gastrocnemius muscle. As shown in Figure 4, the maximal respiration rate in the soleus muscle, a red muscle mainly composed of oxidative fibers, was higher than in the gastrocnemius, which consists mainly of glycolytic fibers. In the soleus, treatment with THP was associated with decreased succinate and TMPD/ascorbate-supported respiration, compatible with an impairment of the complexes II and IV, but possibly also III. In comparison, in gastrocnemius, treatment with THP was associated with impaired TMPD/ascorbate-supported respiration (indicating impaired activity of complex IV), whereas complex I, II and III were not affected.

### Activity of enzyme complexes of the electron transport chain and of citrate synthase

In order to confirm the results obtained by oximetry, we determined the activity of individual enzyme complexes of the electron transport chain in homogenate of soleus and gastrocnemius muscle and related them to the activity of citrate synthase (Table 2). The activity of citrate synthase, a marker of the mitochondrial content (Larsen et al., 2012), was similar in both groups in both muscle types. As shown in Table 2, treatment with THP was associated with a decrease of mitochondrial complex II and IV in soleus, whereas the activity of complex I + III of the electron transport chain were not affected, confirming the results obtained by oximetry. In gastrocnemius, the activities of the enzyme complexes were generally decreased by treatment with THP, but without reaching statistical significance.

**Table 2:** **Enzymatic activities of mitochondrial electron transport chain complexes**.

	**Soleus**			**Gastrocnemius**		
	**CTL**	**THP**	***p*-value**	**CTL**	**THP**	***p*-value**
Complex I + III (relative to CS)	0.020 ± 0.005	0.020 ± 0.006	0.966	0.10 ± 0.02	0.078 ± 0.032	0.561
Complex II (relative to CS)	0.484 ± 0.072	0.166 ± 0.068	0.012	0.218 ± 0.032	0.112 ± 0.040	0.076
Complex IV (relative to CS)	2.43 ± 0.20	1.39 ± 0.26	0.032	0.197 ± 0.048	0.176 ± 0.045	0.751
Citrate synthase (μmol × min^−1^ × g^−1^)	39.1 ± 1.5	40.4 ± 1.4	0.559	12.7 ± 2.1	17.1 ± 3.5	0.294

*Values are expressed as mean ± SEM of 5–6 animals per group for three independent experiments*.

Taken together the results obtained by oximetry and spectrophotometry indicate that carnitine deficiency associated with THP specifically impairs the function of complex II and IV in the oxidative, and of complex IV in the glycolytic muscle.

### H_2_O_2_ production and free radical leak

**Figure 5:**
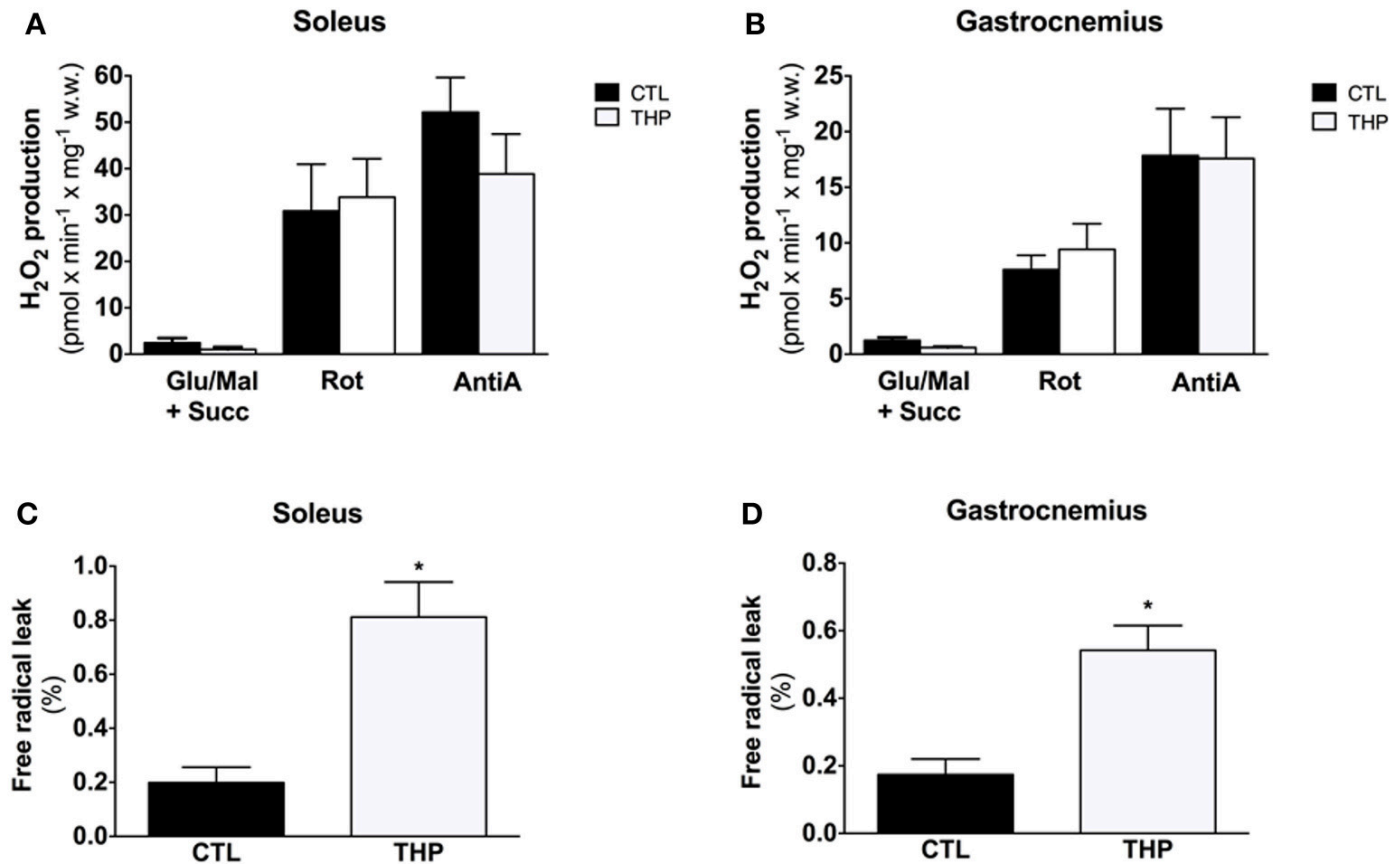
**Mitochondrial H_**2**_O_**2**_ production and Free Radical Leak**. H_2_O_2_ release measured with Amplex Red from permeabilized fibers of **(A)** soleus and **(B)** gastrocnemius and calculated from the slope of change in fluorescence in fibers from rats treated with THP and control rats. FRL calculated as described in the methods for **(C)** soleus and **(D)** gastrocnemius. Data are represented as mean ± SEM of 6 animals per group. ^*^p < 0.05 compared to CTL group.

Figure 5A shows the results of H_2_O_2_ production obtained in permeabilized fibers from the skeletal muscles in response to sequential addition of substrates and inhibitors. Net H_2_O_2_ release by fibers was low in the absence of respiratory substrates in the both treatment groups, but increased by approximately 2-fold after addition of the complex I substrates glutamate and malate. In line with previous observations (Anderson and Neufer, 2006), the highest rates of H_2_O_2_ release under basal conditions were observed in the presence of the complex II substrate succinate. The interruption of normal electron flow with addition of the complex I inhibitor rotenone and of the complex III inhibitor antimycin A was associated a substantial increase in H_2_O_2_ release in both treatment groups. In comparison to CTL rats, H_2_O_2_ production in THP-treated rats was similar under all states investigated for both soleus and gastrocnemius muscle (Figures 5A,B).

The free radical leak (FRL), described as a good index for ROS production (Anderson and Neufer, 2006), was significantly higher in the THP group compared to controls in both muscles in the presence of the complex II substrate succinate (Figures 5C,D). Interestingly, carnitine deficiency enhanced FRL in gastrocnemius muscle (+210%, Figure 5D), whereas the FRL increased robustly in soleus (+309%, Figure 5C) with an increase significantly higher in soleus muscle compared with gastrocnemius.

### SOD1/SOD2 mRNA expression levels and GSH pool in skeletal muscle

**Figure 6:**
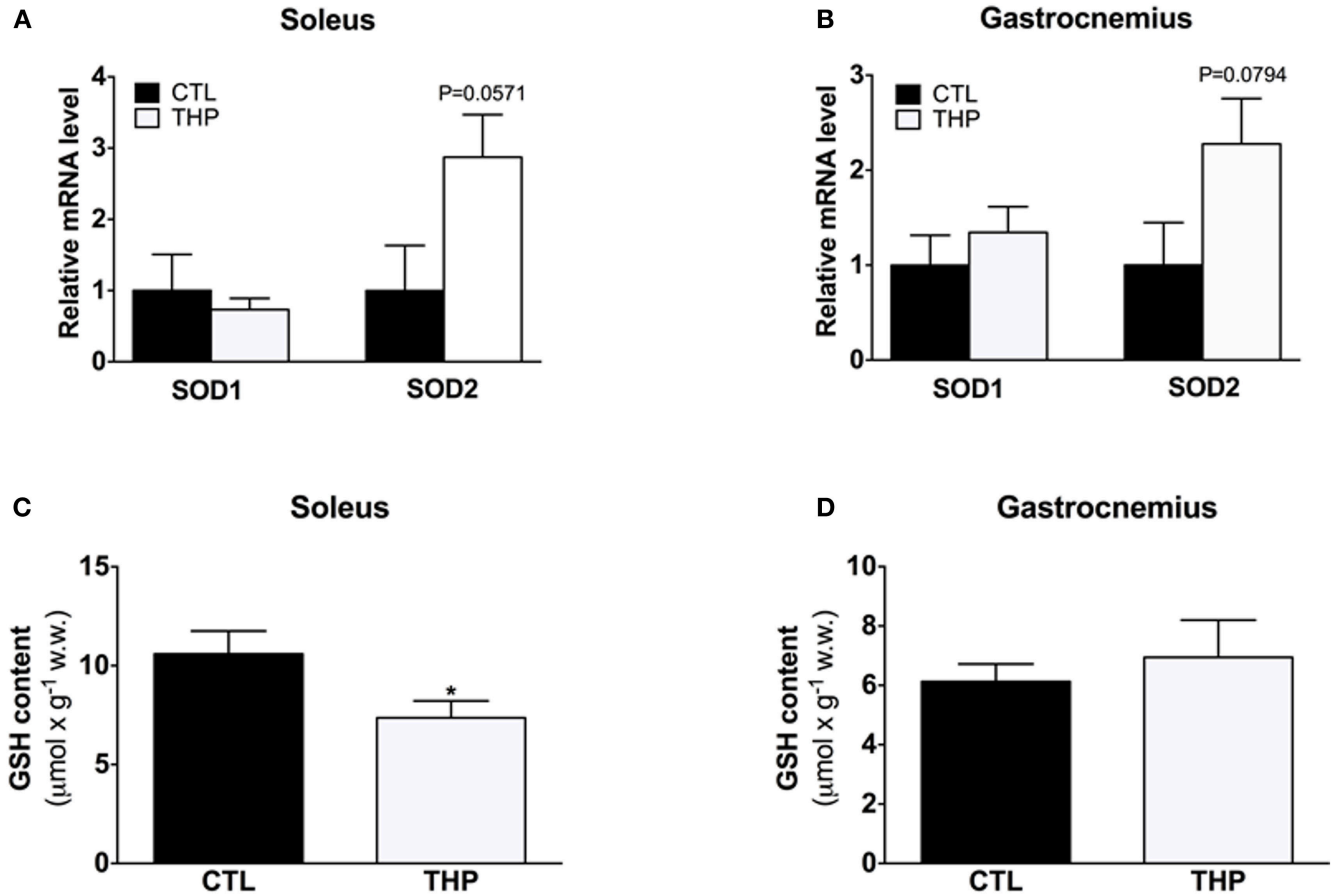
**SOD1/SOD2 mRNA expression and cellular GSH content**. mRNA expression levels of cytosolic SOD1 and mitochondrial SOD2 relative to beta-actin and normalized to control values by q-RT-PCR in **(A)** soleus muscle and **(B)** gastrocnemius muscle of both groups. Reduced GSH was measured in **(C)** soleus muscle and **(D)** gastrocnemius muscle of both groups. Data are represented as mean ± SEM of 6 animals per group. ^*^p < 0.05 compared to CTL group.

To study the antioxidative defense system, we analyzed the mRNA content of cytosolic SOD1 and mitochondrial SOD2 by q-RT-PCR as well as the GSH content in soleus and gastrocnemius muscle. As shown in Figures 6A,B, the SOD1 mRNA expression level was similar in both treatment groups in both muscles. Interestingly, the SOD2 mRNA expression level was increased in soleus of THP-treated as compared to CTL rats, reaching borderline significance (Figure 6A; *p* = 0.057). In gastrocnemius, the SOD2 mRNA expression level was also numerically higher in the THP-group compared to the CTL group (Figure 6B; *p* = 0.0794). The GSH content, which is an important defense against ROS accumulation, was decreased in soleus muscle of THP-treated compared to CTL rats (Figure 6C; *p* < 0.05) but was similar between the treatment groups in gastrocnemius (Figure 6D). Since we did not determine oxidized glutathione (GSSG), this finding could be explained by both increased oxidation of GSH and/or impaired GSH biosynthesis.

### Mitochondrial biogenesis and content

**Figure 7:**
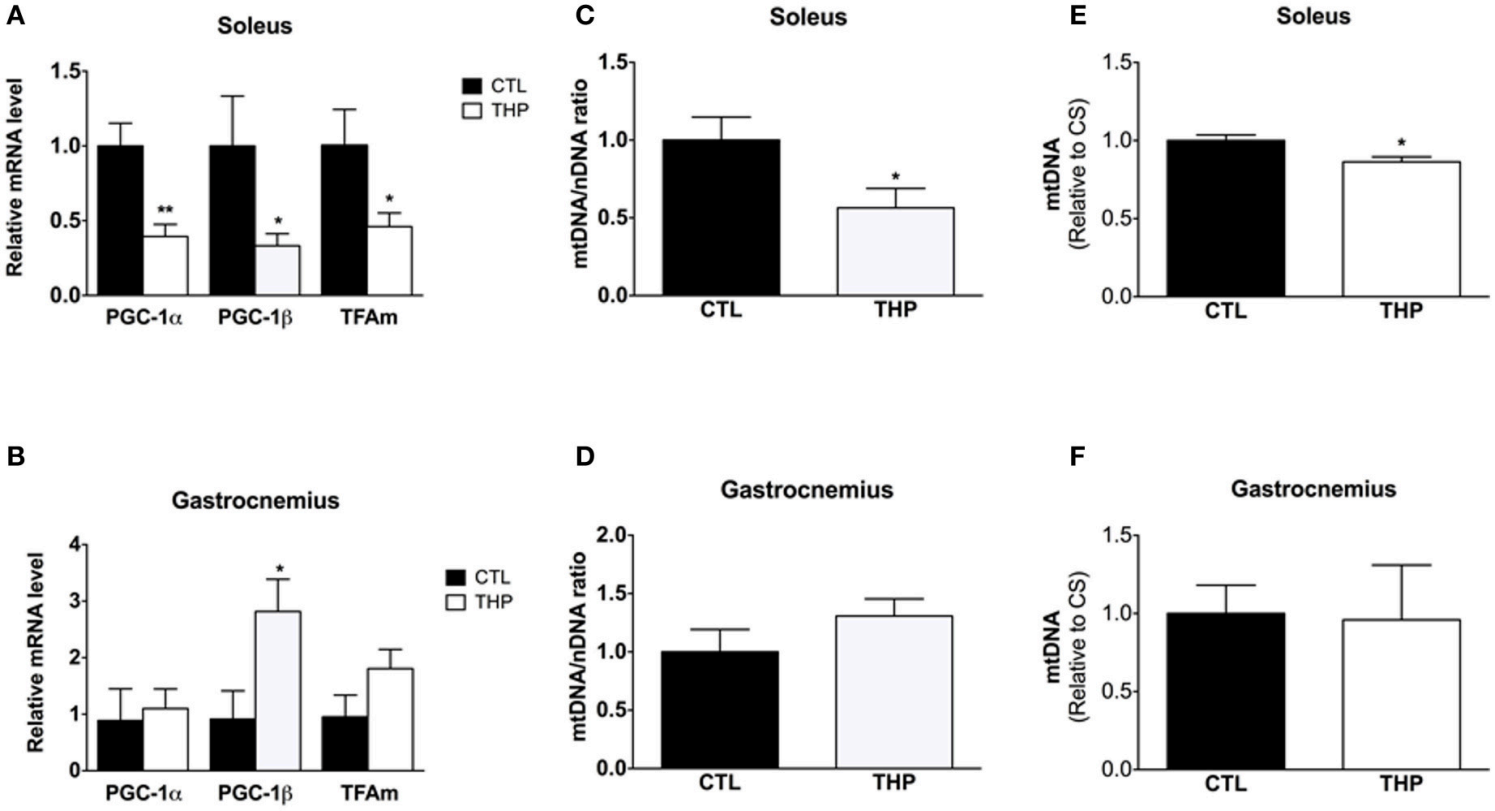
**Mitochondrial biogenesis and DNA content**. mRNA expression of PGC-1α, PGC-1β, and TFAm relative to control values measured by q-RT-PCR in **(A)** soleus and **(B)** gastrocnemius. Mitochondrial to nuclear DNA content of **(C)** soleus and **(D)** gastrocnemius muscle normalized to control values. Mitochondrial DNA content expressed per citrate synthase activity of **(E)** soleus and **(F)** gastrocnemius muscle normalized to control values. Data are represented as mean ± SEM of 6 animals per group. ^*^p < 0.05 and ^**^p < 0.01 compared to CTL group.

Impaired physical performance in THP-treated rats could not only be a consequence of mitochondrial damage, but also of an impaired mitochondrial biogenesis. We therefore determined PGC-1α and PGC-1β mRNA expression levels, both important transcriptional co-activators of numerous genes coding for mitochondrial proteins. mRNA expression of PGC-1α and PGC-1β were lower in the soleus of THP-treated than CTL rats (*p* < 0.01 and *p* < 0.05, respectively; Figure 7A). Consequently, mitochondrial TFAm mRNA expression, which regulates the amount of mtDNA, was also lower in THP-treated compared to the CTL rats (*p* < 0.05; Figure 7A). In contrast, in the gastrocnemius, PGC-1β mRNA expression was increased in THP-treated compared to CTL rats (*p* < 0.05; Figure 7B), whereas the mRNA expression of PGC-1α and of TFAm were not different between the two groups.

In soleus muscle, in agreement with the mRNA expression of PGC-1α and PGC-1β, both the amount of mitochondrial DNA expressed per nuclear DNA (*p* < 0.05; Figure 7C) and per citrate synthase activity (*p* < 0.05; Figure 7E) was decreased in THP-treated compared to control rats. In comparison, in gastrocnemius muscle, the mitochondrial DNA content was not affected by treatment with THP (Figures 7D,F).

## Discussion

The present investigation enlarges our knowledge about the consequences of carnitine depletion due to treatment with THP on exercise performance and mitochondrial function in different types of skeletal muscle in rats. First, we showed that the THP-induced decrease of the muscle carnitine content is associated with impaired exercise capacity. Second, treatment with THP was associated with a marked atrophy of the oxidative soleus muscle as well as with impaired activity of complex II and IV of the electron transport chain, a decrease in the cellular glutathione pool, a decrease in the mRNA expression of regulators of mitochondrial biogenesis, and reduced mitochondrial DNA content compared to control rats. In comparison, in the glycolytic gastrocnemius muscle, THP treatment was associated with only a decrease of complex IV activity, whilst the cellular glutathione pool and the mitochondrial DNA content were not affected compared to control muscle.

In our previous studies, we have shown repeatedly that treatment with THP for 21 days is associated with a 70–80% decrease in the skeletal muscle carnitine content (Spaniol et al., 2001; Zaugg et al., 2003; Roberts et al., 2015). Since we observed a 86% reduction in the plasma total and free carnitine concentrations in THP-treated compared to control rats, and since we reproduced the effect of treatment with THP on the skeletal muscle carnitine pool several times (Spaniol et al., 2001), we did not determine the skeletal muscle carnitine content in the current study. The THP-associated decrease in the skeletal muscle carnitine pool is comparable to the decrease observed in jvs^−/−^ mice (Higashi et al., 2001) or also to humans suffering from primary carnitine deficiency (Shapira et al., 1993).

### Effect of carnitine depletion on exercise capacity

The evaluation of the exercise capacity showed that both the distance covered until exhaustion and vertical mechanical work were reduced in THP-treated rats. Regarding skeletal muscle performance, carnitine has two main functions. First, it is an obligatory intermediate for the transport of long-chain fatty acids across this inner mitochondrial membrane (Fritz and Mc, 1959; Bremer, 1983) and, second, carnitine stimulates the activity of pyruvate dehydrogenase (Wall et al., 2011) by buffering the mitochondrial CoASH pool (Brass and Hoppel, 1980; Friolet et al., 1994). As indicated by the increased lactate blood and skeletal muscle concentrations and by the reduced skeletal muscle glycogen stores, rats exercised above the lactate threshold during the physical performance experiment, suggesting that mainly glucose and to a minor extent fatty acids have been the main fuel for skeletal muscle under these conditions (Gollnick et al., 1974; Roberts et al., 1996). It can therefore be assumed that the effects of carnitine on glucose metabolism have been more important in the current situation than those on fatty acid metabolism. Accordingly, the observed decrease in physical performance is probably mainly due to an impaired function of glycolytic and to a minor extent of oxidative muscles. In a previous study using the same animal model of carnitine deficiency, we have indeed shown in *in vitro* experiments that the maximal contraction of the mainly glycolytic extensor digitorum longus (EDL) muscle is impaired (Roberts et al., 2015). This observation fits well with the current findings. The most probable explanation for this finding was a marked decrease in the EDL muscle free CoA content in rats treated with THP, leading to a reduced flux through pyruvate dehydrogenase (Roberts et al., 2015). In the current studies, we additionally found a significant impairment in the activity of complex IV in the gastrocnemius muscle of THP treated rats, offering an additional explanation regarding the observed reduction in exercise capacity. Furthermore, we found an increased free radical leak, possibly as a consequence of the impaired activity of the mitochondrial electron transport chain (Balaban et al., 2005). The cellular glutathione content was not affected in gastrocnemius muscle of THP-treated rats, suggesting that the increase of the free radical leak was not large enough to impair the antioxidative defense system.

### Effect of carnitine depletion on oxidative skeletal muscle

In comparison to the glycolytic gastrocnemius muscle, the effects of carnitine depletion were more pronounced on the oxidative soleus muscle. Nevertheless, as discussed above, these findings may contribute, but cannot entirely explain the observed decrease in exercise performance in THP-treated rats, since the rats were exercising above the lactate threshold. Similar to the gastrocnemius, carnitine depletion was associated with increased lactate content after exercise in the soleus muscle. Since, in our previous study (Roberts et al., 2015), the free CoA content was only numerically decreased and the *ex vivo* pyruvate dehydrogenase activity maintained in soleus muscle of THP-treated rats, this finding is explained best by the observed impairment of complex II and IV of the electron transport chain, which was more pronounced in the oxidative soleus compared to the glycolytic gastrocnemius muscle. Impaired activity of the electron transport chain is associated with an increase in the mitochondrial NADH/NAD ratio, which could *in vivo* impair the activity of pyruvate dehydrogenase (Patel et al., 2014). Similar to the gastrocnemius muscle, we observed an increase in ROS production relative to oxygen used (increased free radical leak), which was associated with an increase in the mRNA expression of SOD2 and with a decrease in the cellular GSH content. A possible interpretation of these findings is that the ROS production in soleus muscle was high enough to reduce the cellular glutathione pool and to induce SOD2 mRNA expression. Increased mRNA expression of SOD2 as a consequence of enhanced ROS production has been described previously (Kensler et al., 2007; Felser et al., 2013). Since carnitine has been described to possess antioxidative properties (Gülçin, 2006), carnitine deficiency could decrease the antioxidative capacity, leading to oxidative stress already at normally tolerated ROS production levels.

Increased ROS production has been described not only to up-regulate the antioxidative defense system including the Nrf2 pathway, and SOD2 (Kensler et al., 2007), but also to stimulate mitochondrial proliferation (Piantadosi and Suliman, 2012). Accordingly, in the gastrocnemius muscle, the mRNA expression of mitochondrial proliferation regulators (PGC-1α, PGC-1β, TFAm) was numerically increased, with PGC-1β reaching statistical significance. This increase in the mRNA expression of mitochondrial proliferation regulators did, however, not translate into a significant increase in mtDNA. In contrast, in the oxidative soleus muscle, which is rich in mitochondria, mRNA expression of the above mentioned regulators of mitochondrial proliferation was significantly decreased in THP-treated rats with a consequent significant decrease in the mtDNA related to nuclear DNA or citrate synthase activity. The reasons for the different reaction of the oxidative soleus vs. the glycolytic gastrocnemius muscle to carnitine depletion are currently not clear, but may be related to the more accentuated mitochondrial damage and to muscle atrophy that we observed in soleus muscle in the current as well as in our previous study (Roberts et al., 2015). In our previous study, we could demonstrate that soleus muscle atrophy in THP-treated rats is a consequence of apoptosis, which is triggered most likely by mitochondrial dysfunction (Roberts et al., 2015).

## Conclusions

The current study showed that the THP-induced carnitine deficiency was associated with a decreased physical performance in rats exercising above the lactate threshold. Carnitine depletion affected oxidative and glycolytic muscles differently. In the oxidative soleus, we found muscle atrophy and reduced activity of complex II and IV of the electron transport chain with a concomitant increase in ROS production and alterations in the antioxidative defense system. mRNA expression of mitochondrial proliferation markers and mtDNA were reduced. In comparison, in the glycolytic gastrocnemius, muscle mass was not decreased, only complex IV of the electron transport chain was impaired and the oxidative defense system as well as the mtDNA content were not affected. Taking into account that glucose is the main fuel for muscle work above the lactate threshold, the observed decrease in physical performance of THP-treated rats can be explained by an impaired buffer function of carnitine regarding free CoASH and by the observed mitochondrial dysfunction in glycolytic and oxidative muscles.

## Author contributions

JB, SK developed the research idea and experimental design. JB, PH, FS, LJ, AF, and UD conducted experiments, collected and analyzed data. JB, SK wrote the manuscript and all authors read, corrected and approved the final manuscript.

## Funding

This research received a grant from the Swiss National Science Foundation (SNF 31003A-156270) to SK.

### Conflict of interest statement

The authors declare that the research was conducted in the absence of any commercial or financial relationships that could be construed as a potential conflict of interest.

## Abbreviations

FRL: Mitochondrial Free Radical Leak
GSH: Reduced glutathione
Nrf-2: Nuclear factor erythroid 2–related factor 2
OCTN2: Organic Cation/Carnitine Transporter 2
PGC: Peroxisome proliferator-activated receptor gamma coactivator
SOD: Superoxide dismutase
TFAm: Mitochondrial transcription factor A
THP: N-trimethyl-hydrazine-3-propionate.
